# Neu5Ac Induces Human Dental Pulp Stem Cell Osteo-/Odontoblastic Differentiation by Enhancing MAPK/ERK Pathway Activation

**DOI:** 10.1155/2021/5560872

**Published:** 2021-09-23

**Authors:** Changzhou Li, Xinghuan Xie, Zhongjun Liu, Jianhua Yang, Daming Zuo, Shuaimei Xu

**Affiliations:** ^1^Department of Medical Laboratory, School of Laboratory Medicine and Biotechnology, Southern Medical University, Guangzhou, Guangdong 510515, China; ^2^Department of Immunology, School of Basic Medical Sciences, Southern Medical University, Guangzhou, China; ^3^Department of Endodontics, Stomatological Hospital, Southern Medical University, Guangzhou, China; ^4^Department of Orthopaedics, Longgang District People's Hospital of Shenzhen, Shenzhen, China; ^5^Microbiome Medicine Center, Department of Laboratory Medicine, Zhujiang Hospital, Southern Medical University, Guangzhou, China

## Abstract

Dental pulp stem cells (DPSCs) must undergo odontoblastic differentiation in order to facilitate the process of dentin-pulp complex repair. Herein, we sought to explore the ability of Neu5Ac (one form of sialic acid) to influence DPSC osteo-/odontoblastic differentiation via modulating mitogen-activated protein kinase (MAPK) signaling. *Methodology*. DPSCs were isolated from human third permanent teeth and were grown *in vitro.* Fluorescent microscopy was used to detect the existence of sialic acid on the DPSC membrane. Following the treatment of different concentrations of Neu5Ac and removing sialic acid from the cell surface by neuraminidase, the osteo-/odontoblastic differentiation of these cells was evaluated via mineralization, alkaline phosphatase, and *in vivo* assays. In addition, the expression of genes related to osteo-/odontoblastic differentiation and MAPK signaling at different stages of this differentiation process was analyzed in the presence or absence of Neu5Ac. *Results*. The existence of sialic acid on the DPSC membrane was confirmed by fluorescent microscopy, and the ability of osteo-/odontoblastic differentiation was decreased after removing sialic acid by neuraminidase. Treatment of DPSCs with Neu5Ac (0.1 mM or 1 mM) significantly enhanced their mineralization ability and alkaline phosphatase activity. The expression levels of DMP1, DSPP, BSP, and RUNX2 were also increased. Treatment of nude mice with ManNAc (the prerequisite form of Neu5Ac) also enhanced DPSC mineralization activity *in vivo.* Furthermore, Neu5Ac treatment enhanced p-ERK expression in DPSCs, while ERK pathway inhibition disrupted the ability of Neu5Ac to enhance the osteo-/odontoblastic differentiation of these cells. *Conclusions*. Neu5Ac can promote DPSC osteo-/odontoblastic differentiation through a process associated with the modulation of the ERK signaling pathway activity.

## 1. Introduction

Stem cells from oral cavity are easily harvestable and have shown a great plasticity towards the main lineages, specifically towards bone tissues [1]. Dental pulp stem cells (DPSCs) are the most investigated and commonly evaluated in the context of tissue engineering and regenerative medicine [2]. Their clinical utility, however, is limited by the fact that relatively few of these cells are available, and they lose their ability to differentiate over the course of *in vitro* expansion [3]. Enhancing the utility of these cells in clinical tissue engineering contexts will therefore necessitate the development of novel approaches to improving DPSC osteo-/odontoblastic differentiation capacity. Many factors to date have been found to impact this differentiation capacity, such as proinflammatory cytokines [4], growth factors [5], mechanical stretch, bioscaffolds/biomaterials [6, 7], and donor age [8]. Previous studies suggested that tissue inflammation may act against the tooth/bone formation/repair, and some natural compounds may be useful to alleviate this effect [9, 10]. These findings underscore the fact that the external microenvironment is a key determinant of DPSC fate, indicating that accurately recapitulating these conditions may represent a viable approach to apply these cells in the context of tissue regeneration.

Sialic acid (SA) is a component of cell surface sugar moieties that are associated with N- and O-glycan chains and glycolipids wherein it can be attached via *α*2-3, *α*2-6, and *α*2-8 linkages [11]. SA expression is evident across vertebrate and nonvertebrate species and in mammals. It was primarily found in two major forms: N-acetylneuraminic acid (Neu5Ac) and N-glycolylneuraminic acid (Neu5Gc) [12]. Of these forms, only Neu5Ac is found in healthy humans [13], wherein it serves as an important regulator of interactions between cells and of signaling, enzymatic, and antibody-related activities [14].

The functional roles of sialic acid in many experimental contexts are well understood. Li et al. explored its role in the context of tumor cell proliferation and migration by removing sialic acid from the AGS gastric cancer cell line [15]. This led these authors to discover that MAL-II could specifically recognize and interact with terminal sialic acid residues within glycoprotein chains. Furthermore, they found that the treatment of AGS cells with *α*2,3-neuraminidase, which cleaved cell surface sialic acid, enhanced the repair and migratory capabilities of these cells, highlighting the ability of sialic acid to drive cell-related signaling and behavior. Xu et al. further found that reductions in the SA expression on an osteoblast cell line were associated with decreases in both bone mineralization and the expression of bone sialoprotein (BSP), osteoprotegerin (OPG), and vitamin D receptor, indicating that SA plays key roles in the context of osteogenesis [16]. SA is closely related to many oral diseases such as recurrent aphthous ulcer, oral potentially malignant disorders (OPMD), and oral cancers [17]. Patients with OPMD and oral cancers exhibit a high SA concentration in the serum and saliva [17], while patients with recurrent aphthous stomatitis and other oral inflammations exhibit a low concentration of sialic acid [18]. As a kind of mesenchymal stem cells, DPSC has a great potential in oral clinical treatment. The specific role played by sialic acid in the context of DPSC osteo-/odontoblastic differentiation, however, has yet to be explored.

The present study was thus designed to assess whether Neu5Ac treatment was sufficient to enhance DPSC osteo-/odontoblastic differentiation, and if so, what signaling pathways and molecular mechanisms underlie such enhancement.

## 2. Methodology

### 2.1. DPSC Isolation and Culture

Caries-free third molars from 10 healthy patients (20–24 years old) were collected immediately following the extraction and were used to isolate DPSCs. Dental pulp was isolated under sterile conditions and rinsed with PBS, after which they were minced using a ophthalmological scissors, and pulp aliquots were transferred into 6-well plates containing general medium (GM) composed of *α*-MEM containing 10% FBS and 1% penicillin-streptomycin (Gibco, USA). Cells were cultured in a 37°C with 5% CO_2_ incubator, with media being changed every other day until cells reached confluence, at which time cells were passaged. Cells were used for experimentation following 3-5 passages. Osteo-/odontoblastic differentiation was induced in GM supplemented with 50 mg/ml ascorbic acid, 10 mM *β*-glycerol phosphates, and 10^−7^ mol/l dexamethasone. DPSC cell surface expression of stem cell markers (CD29, CD31, CD34, CD44, CD45, CD90, and CD105) was assessed via flow cytometry using antibodies from BD Biosciences (USA). The ethics committee of the Stomatological Hospital of Southern Medical University approved this study.

### 2.2. Treatment of DPSC with Neuraminidase or Neu5Ac

To evaluate the role of cell surface sialic acid in the odontoblastic differentiation process, DPSCs were treated with Neu5Ac or neuraminidase, which desialylated cell surface glycoconjugates. The impact of Neu5Ac on DPSCs was assessed by treating them with 0 mM, 0.1 mM, or 1 mM of Neu5Ac (Sigma-Aldrich, St Louis, MO, USA) added to the cell culture media. With regard to neuraminidase, DPSCs were exposed to neuraminidase (0 mU/ml, 1 mU/ml, 10 mU/ml, or 100 mU/ml, Sigma-Aldrich).

### 2.3. Cell Viability Assay (CCK-8 Assay)

The cell viability was detected by Cell Counting Kit-8 (CCK-8) (Dojindo Laboratories, Kumamoto, Japan). Briefly, the cells were exposed to 0 mM, 0.1 mM, or 1 mM of Neu5Ac or 0 mU/ml, 1 mU/ml, 10 mU/ml, or 100 mU/ml of neuraminidase in 96-well plates for 1, 3, 5, or 7 days, and six wells were prepared for each dose of Neu5Ac and neuraminidase. After treatment, 10 *μ*l of CCK-8 solution was added to each well, and the 96-well plate was continuously incubated at 37°C for 1 hour; then, the OD value for each well was read at wavelength 450 nm to determine the cell viability on a microplate reader (Multiskan, Thermo, USA).

### 2.4. qRT-PCR

Cells were plated at 2 × 10^5^ cells/well and were treated for 4, 7, or 14 days with neuraminidase (0 mU/ml, 1 mU/ml, 10 mU/ml, or 100 mU/ml) or Neu5Ac (0 mM, 0.1 mM, or 1 mM), after which TRIzol (Invitrogen, CA, USA) was used to extract total cell RNA based on provided instructions. A First-Strand cDNA Synthesis Kit (Gibco, USA) was then used to prepare cDNA from 1 g of total RNA per sample, after which qRT-PCR was conducted with SYBR green (Takara, Japan) and a thermocycler instrument (ABI7500, Applied Biosystems, USA). The 2^-*ΔΔ*Ct^ method was employed to evaluate relative gene expression, with GAPDH being used as a normalization control. Primers used in this analysis are shown in Table 1.

### 2.5. Western Blotting

Western blotting was used to assess the MAPK signaling pathway and odontoblastic differentiation-related protein expression in DPSCs that were (1) treated with neuraminidase (0, 1, 10, or 100 mU/ml) for 4 days; (2) treated with Neu5Ac (0, 0.1, or 1 mM) for 4, 7, or 14 days; (3) treated with Neu5Ac (1 mM) for 0, 10, 30, 60, 90 minutes or for 3 days; and (4) pretreated with the extracellular signal-related kinase (ERK) inhibitor cobimetinib (1 *μ*M) for 4 hours and then treated with or without Neu5Ac (1 mM) for 4 days.

Following the above treatments, DPSCs were washed twice with PBS before resuspension in lysis buffer (Cell Signaling Technology, MA, USA) supplemented with phenylmethylsulfonyl fluoride (R&D Systems, Minneapolis, MN, USA) to facilitate protein extraction. A BCA assay was used to quantify protein levels in these extracts, after which samples were separated via SDS-PAGE and transferred to PVDF membranes. Blots were blocked for 1 hour with 5% nonfat milk, after which they were probed overnight with anti-DSPP, anti-DMP1, anti-RUNX2, anti-phospho-ERK, anti-ERK, anti-phospho-p38, anti-p38, anti-phospho-JNK, or anti-JNK (Cell Signaling Technology, MA, USA) at 4°C. Blots were then probed with appropriate secondary antibodies for 1 hour, after which protein bands were detected via a chemiluminescent approach.

### 2.6. ALP Staining

DPSCs were initially cultured in 6-well plates (1 × 10^5^/well) in the presence or absence of Neu5Ac or neuraminidase for 7 days. Culture media were then removed, and cells were fixed for 1 hour with 70% ethanol, after which they were stained with 300 *μ*l of ALP staining reagent (1-Step NBT/BCIP solution, Thermo Fisher Scientific) per well. Water was then added to terminate staining, and the stain was extracted via the addition of 10% (*w*/*v*) cetylpyridinium chloride (Sigma-Aldrich) for 15 minutes. Staining intensity was then quantified using a VERSA max Multiplate Reader by assessing absorbance at 540 nm.

### 2.7. Alizarin Red Staining

DPSCs were initially grown for 21 days in the presence of neuraminidase, Neu5Ac and/or cobimetinib, after which culture media were removed and cells were rinsed with PBS. Cells were then fixed for 1 hour using 70% chilled ethanol, followed by staining for 15 minutes with 40 mM Alizarin S (pH 4.2) at room temperature with gentle stirring. Cells were then washed five times using PBS, after which staining was quantified via extracting the stain for 15 minutes with 10% (*w*/*v*) cetylpyridinium chloride (Sigma-Aldrich, St. Louis, MO, USA) and evaluating absorbance at 540 nm with a VERSA max Multiplate Reader.

### 2.8. Fluorescent Microscopy

The existence of sialic acid on the DPSC membrane was detected as described previously [19]. Neuraminidase-treated (100 mU/ml, 3 hour) and untreated DPSCs were washed with PBS three times, fixed with 4% paraformaldehyde for 30 minutes at room temperature (RT), rinsed with PBS for three times, and then blocked with 3% bovine serum albumin (BSA; Solarbio) for 1 hour. Washed with PBS three times, incubated with 10 *μ*g/ml fluorescein isothiocyanate- (FITC-) labeled lectin A. hypogaea (PNA) (Sigma-Aldrich, St. Louis, MO, USA) overnight in a moist chamber at 4°C. PNA can bind to the galactose moiety exposed on cell surface glycoconjugates after removing the terminal sialic acid. The next day, samples were incubated with DAPI (1 : 200) for 15 minutes at room temperature. Fluorescence microscopy images were captured under a fluorescence microscope (IX71 FL, Olympus).

### 2.9. In Vivo Osteogenesis Assay

*In vivo* ectopic osteogenesis was evaluated by subcutaneously implanting passage 3 DPSCs that had been mixed with 40 mg of 1.0 mm hydroxyapatite/*β*-tricalcium phosphate (HA/*β*-TCP) particles (ratio 3 : 8; Sichuan University Biomaterials Engineering Research Center, Chengdu, China) into the backs of nude mice (BALB/c, 6-weeks-old; Bianco, Kuznetsov, Riminucci, & Gehron Robey, 2006). A total of 8 mice were randomly divided into two groups with 4 mice per group; each group contains 2 female mice and 2 male mice. Mice were gavaged for 6 consecutive weeks with ManNAc (2 g/kg/animal/day) every day. The reason for choosing ManNAc over Neu5Ac is because ManNAc is the prerequisite form of Neu5Ac and can only transform to Neu5Ac in the animal organism. In addition, the absorption of ManNAc *in vivo* is better than that of Neu5Ac [20], beginning on day 2 following implantation. Control animals were administered PBS in lieu of ManNAc every day. Following the 6-week treatment period, the transplanted region was fixed, demineralized with 10% EDTA solution for 7 days, paraffin-embedded, and cut into 2 *μ*m sections that were then subjected to hematoxylin and eosin staining. DSPP and RUNX2 were then detected via immunohistochemical (IHC) staining with an appropriate antibody (1 : 50; ab122321; Abcam). The immunostained images were analyzed and scored by two pathologists independently in a blinded manner, based on the *H*-score method, which considers the percentage of cells staining positively together with the staining intensity [21, 22]. 10 fields at 400x magnification were chosen randomly. The staining intensity of weak, intermediate, and strong staining was scored as 0, 1, 2, and 3, corresponding to the negative control. The total number of cells and cells stained at each intensity were counted in each field. The *H*-score was calculated according to the formula: (%of cells stained at intensity category 1 × 1) + (%of cells stained at intensity category 2 × 2) + (%of cells stained at intensity category 3 × 3). *H*-scores ranged from 0 to 300 where 300 indicated 100% of cells strongly stained (3+). *H*-scores of cells ≥200 were defined as a high expression. The Ethics Committee of Southern Medical University approved these animal studies. *In vivo* cell viability assay, KI67 was detected via immunohistochemical (IHC) staining with an appropriate antibody (1 : 50; ab122321; Abcam). The immunostained images were analyzed and scored by two pathologists independently in a blinded manner based on the *H*-score method which has mentioned above.

### 2.10. Statistical Analysis

Experiments were conducted in triplicate, and data are means ± SD. SPSS v17.0 was used for all statistical testing. Data were statistically analyzed using Student's *t*-test, with *p* < 0.05 as the significance threshold. All graphs were plotted using GraphPad Prism 8 (GraphPad Software, Inc., La Jolla, CA, USA).

## 3. Results

### 3.1. DPSC Identification

**Figure 1:**
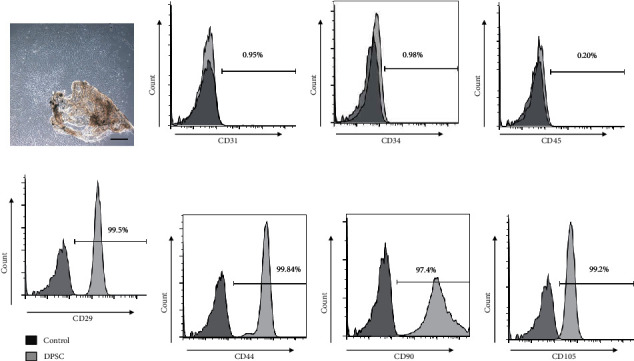
Primary human DPSC identification. (a) Primary DPSCs were isolated from dental pulp tissue. (b–h) Isolated DPSCs were CD29, CD44, CD90, and CD105 positive and were CD31, CD34, and CD45 negative in flow cytometry analyses. All the experiments were repeated at least three times independently. Scale bar = 100 *μ*m.

We first validated the identity of the DPSCs used in the present study via flow cytometry. This analysis confirmed that these cells were positive for mesenchymal stem cell markers CD29, CD44, CD90, and CD105 while negative for the hematopoietic stem cell markers CD31, CD34, and CD45. These results suggested that we successfully isolated and cultured the DPSCs (Figure 1).

### 3.2. Desialylation of DPSC by Neuraminidase Inhibits Osteo-/Odontoblastic Differentiation of DPSC

**Figure 2:**
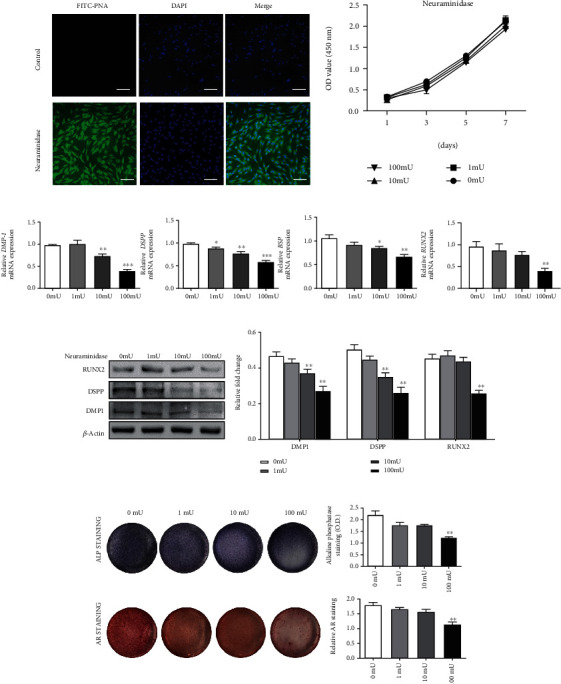
The effect of neuraminidase on DPSC osteo-/odontoblastic differentiation. (a) FITC-PNA lectin staining of DPSCs with or without neuraminidase (100 mU/ml) treatment. (b) CCK-8 assay results of neuraminidase-treated DPSC. (c) mRNA expression of DMP1, DSPP, BSP, and RUNX2 in DPSCs treated with neuraminidase (0, 1, 10, and 100 mU/ml) for 4 days. (d) Protein expression of DSPP, DMP1, and RUNX2 in DPSCs treated with neuraminidase (0, 1, 10, and 100 mU/ml) for 4 days. (e) ALP activity and Alizarin red staining after DPSCs were treated with neuraminidase (0, 1, 10, and 100 mU/ml). Scale bars = 50 *μ*m. ^∗^*p* < 0.05, ^∗∗^*p* < 0.01, and ^∗∗∗^*p* < 0.001. All the experiments were repeated at least three times independently.

FITC-PNA fluorescent staining was used to detect the existence of sialic acid on the DPSC surface. We observed no FITC-PNA fluorescent staining in untreated DPSCs, while FITC-PNA fluorescent staining was significant enhanced in neuraminidase-treated DPSCs (Figure 2(a)). This proved that neuraminidase can effectively remove the sialic acid on the DPSC surface and indirectly proved the existence of sialic acid on the DPSC membrane. After we treated DPSC with different concentrations (0, 1, 10, and 100 mU/ml) of neuraminidase for 1, 3, 5, or 7 days, the CCK-8 assay showed that there was no significant difference in the OD value of each group of cells, indicating that the concentrations of neuraminidase used in this study had no significant effect on the viability of DPSC (Figure 2(b)). Removing sialic acid from the DPSC membrane by neuraminidase reduced the mRNA expression levels of osteo-/odontogenic markers DMP1, DSPP, BSP, and RUNX2 (Figure 2(c)). Western blot analysis showed that neuraminidase also inhibited the protein levels of RUNX2, DMP1, and DSPP (Figure 2(d)). Consistent with the results of protein and mRNA expressions, neuraminidase could reduce the staining of ALP and Alizarin red, as indicated that neuraminidase could inhibit the formation of calcified nodules (Figure 2(e)). Together, these results suggested that DPSC odontoblastic differentiation was decreased in the absence of Neu5Ac.

### 3.3. The Impact of Neu5Ac on DPSC Odontoblastic Differentiation

**Figure 3:**
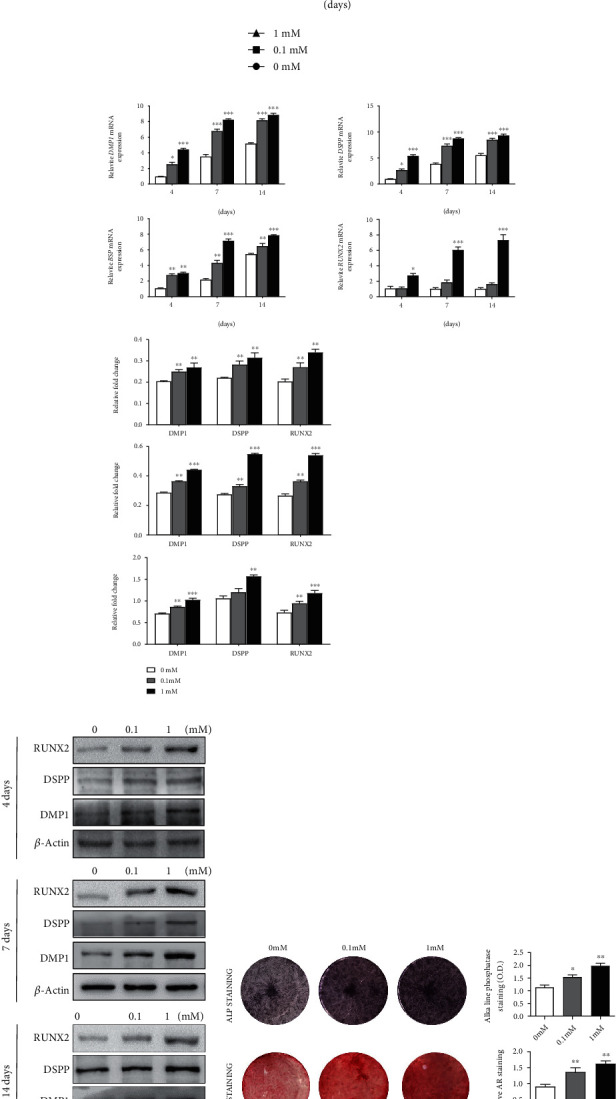
The impact of Neu5Ac on DPSC osteo-/odotoblastic differentiation. (a) CCK-8 assay results of Neu5Ac-treated DPSC. (b) qRT-PCR detected the expressions of DMP1, DSPP, BSP, and RUNX2 after treating DPSCs with Neu5Ac (0.1 or 1 mM) for 4, 7, or 14 days, with GAPDH used as a normalization control. (c) DMP1, DSPP, and RUNX2 protein expressions were detected by western blotting. (d) The ability of DPSC with Neu5Ac treatment (0.1 and 1 mM) assessed via ALP activity and Alizarin red staining. Data are means ± SD. ^∗^*p* < 0.05, ^∗∗^*p* < 0.01, or ^∗∗∗^*p* < 0.001. All the experiments were repeated at least three times independently.

In order to evaluate the impact of Neu5Ac on DPSC odontoblastic differentiation, we firstly treated DPSC with different concentrations (0, 0.1, or 1 mM) of Neu5Ac for an indicated time point, and the CCK-8 assay showed that the concentrations of Neu5Ac used in this study had no significant effect on the viability of DPSC (Figure 3(a)). Next, we assessed the expression of key osteo-/odontogenic marker genes in cells. The data revealed that Neu5Ac treatment was associated with significant increases in the mRNA expressions of DSPP, DMP1, BSP, and RUNX2 (Figure 3(b)). This was further supported by the protein level expressions of DMP1, DSPP, and RUNX2 in Neu5Ac-treated cells (Figure 3(c)).

**Figure 4:**
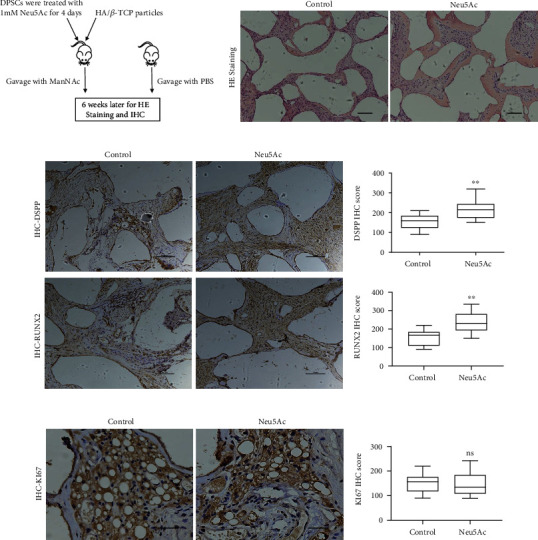
The method and impact of the ManNAc on DPSCs *in vivo*. (a) Flow chart of *in vivo* ectopic osteogenesis of DPSCs. (b) Hematoxylin and eosin stain showed enhanced osteogenesis in the ManNAc group than in the control group. (c, d) Immunohistochemistry and statistical analysis of the average *H*-score showed the effect of ManNAc on proliferation and differentiation of DPSCs. Scale bar = 100 *μ*m, ^∗∗^*p* < 0.01. All the experiments were repeated at least three times independently.

We additionally observed clear evidence of dose-dependent enhancement of ALP activity in Neu5Ac-treated cells on day 7 after the induction of osteo-/odontoblastic differentiation. Additionally, Alizarin red staining conducted on day 14 was used to evaluate the impact of Neu5Ac on mineralization activity. The result exhibited enhanced mineralization activity in response to Neu5Ac treatment at this time point compared to control treatment (Figure 3(d)). Furthermore, we conducted *in vivo* osteogenesis assays and observed enhanced osteogenesis in ManNAc-treated mice compared to control animals, determined by HE staining and IHC staining with DSPP and RUNX2. Notably, KI67 immunohistochemistry showed no significant difference in cell viability between the two groups (Figure 4). Taken together, *in vitro and in vivo* experiments indicated that Neu5Ac positively correlated with the osteo-/odontoblastic differentiation of DPSCs.

### 3.4. Neu5Ac Activates ERK Signaling to Drive DPSC Mineralization

**Figure 5:**
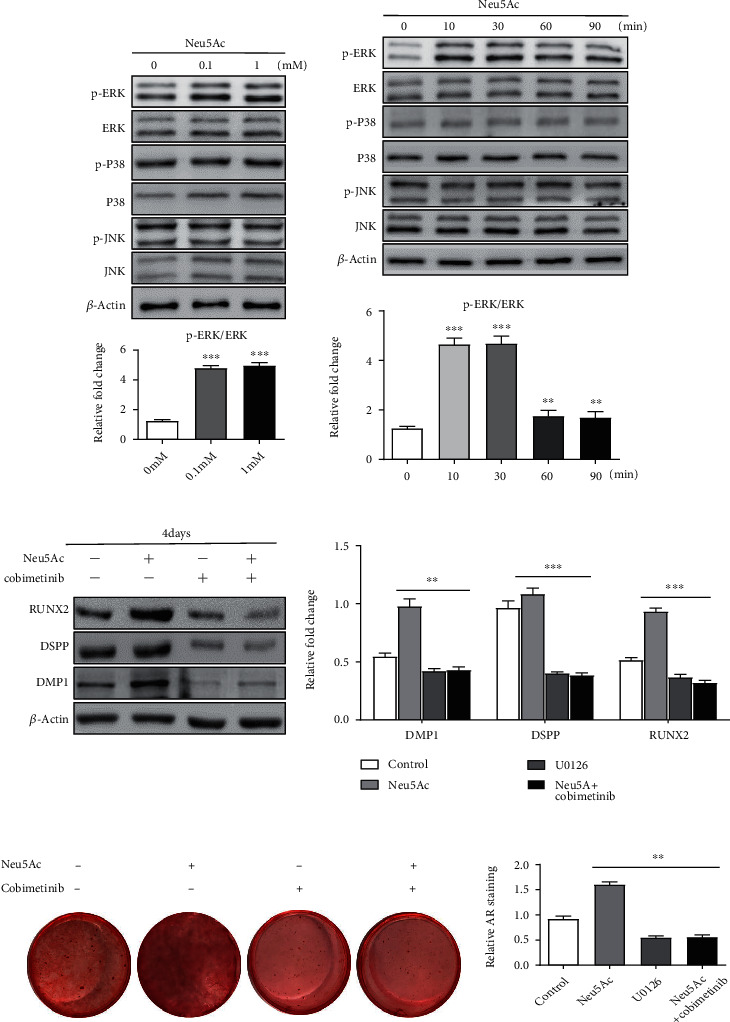
Neu5Ac enhances DPSC osteo-/odontoblastic differentiation via activating ERK signaling. (a) ERK, p-ERK, p38, p-p38, JNK, and p-JNK levels in DPSCs were evaluated following a 3-day treatment with Neu5Ac (0, 0.1, or 1 mM) via Western blotting. (b) The p-ERK/total ERK, p-p38/total p38, and p-JNK/total JNK ratios were determined for DPSCs following treatment with 1 mM Neu5Ac for 0, 10, 30, 60, or 90 minutes via Western blotting. (c) DMP1, DSPP, and RUNX2 protein levels in DPSCs treated with either Neu5Ac and/or cobimetinib were measured. (d) Alizarin red staining of DPSCs was conducted following a 14-day treatment period with cobimetinib and/or Neu5Ac. ^∗∗^*p* < 0.01, ^∗∗∗^*p* < 0.001. All the experiments were repeated at least three times independently.

Lastly, we evaluated the relationship between the MAPK signaling pathway and Neu5Ac-mediated enhancement of DPSC osteo-/odontoblastic differentiation. We observed no changes in the protein level expressions of total ERK, JNK, or p38 following Neu5Ac treatment. Interestingly, we observed significant increases in p-ERK levels that peaked at 30 minutes post-Neu5Ac treatment and remained elevated for at least 90 minutes. Even on day 3 posttreatment, the p-ERK/ERK ratio was obviously higher for cells treated with Neu5Ac (0.1 and 1 mM) relative to that in untreated control cells (Figures 5(a) and 5(b)). No changes in p-JNK or p-p38 levels were detected throughout treatment. To evaluate the role of ERK pathway signaling on Neu5Ac-mediated enhancement of DPSC mineralization, we next pretreated DPSCs with the ERK inhibitor cobimetinib. This analysis revealed that ERK inhibition abrogated the Neu5Ac-induced upregulation of DMP1, DSPP, and RUNX2 in these DPSCs (Figures 5(c) and 5(d)). These findings indicated that Neu5Ac influences DPSC differentiation mainly via the ERK pathway.

## 4. Discussion

In recent years, with the in-depth studies of stem cells, many types of stem cells have shown their clinical therapeutic potential [23–27]. The stable and reliable sources of stem cells is particularly important [28]. DPSCs can be readily isolated following the extraction of healthy teeth, and these cells are highly amenable to proliferating and differentiating into osteo-/odontoblasts [29], making them ideally suited to use in bone and dental tissue engineering. However, the clinical applicability of these cells has been limited to date because they rapidly lose their ability to proliferate and undergo multipotent differentiation throughout prolonged *in vitro* culture [30]. Therefore, it is vital that novel strategies capable of stimulating prolonged DPSC proliferation and differentiation be developed. As such, we herein evaluated the ability of Neu5Ac to enhance DPSC odontoblastic differentiation. To determine whether there is SA on the surface of DPSCs, we firstly assumed that there was SA on the surface of the cells. After treatment with neuraminidase, FITC-PNA fluorescent staining was significantly enhanced. This proved that neuraminidase can effectively remove the sialic acid on the DPSC surface and indirectly proved the existence of sialic acid on the DPSC membrane.

ALP activity and expression are an early indicator of osteoblastogenesis [31], whereas Alizarin red staining can reliably detect mineralized nodules [32]. We found that treating DPSCs with Neu5Ac was sufficient to enhance both ALP activity and the formation of mineralized nodules. We also evaluated the expression of key odontoblastic differentiation-related genes, including the dentin-specific DSPP [33], and the osteogenesis marker genes DMP1, BSP, and RUNX2 [34]. We observed clear increases in DSPP, DMP1, and RUNX2 protein levels as well as BSP mRNA level in DPSCs that had been treated with Neu5Ac, thus emphasizing the ability of Neu5Ac to enhance the osteo-/odontoblastic differentiation of these cells.

The results of the present study suggested that sialic acid played an important role in DPSC odontoblast differentiation. Removing SA from cell surface appeared to strongly inhibit odontoblast differentiation, while treatment with a high concentration of Neu5Ac presented the opposite tendency. These data indicated that SA was involved in the DPSC odontoblast differentiation process. Considering that SA played an important role in the cell-cell adhesion process [35, 36], SA may influence the DPSC odontoblast differentiation through cell-cell fusion.

MAPK are cytoplasmic serine/threonine kinases that are universally expressed in mammalian cells. Many cytokines and other stimuli can induce ERK1/2 activation, thereby modulating cellular proliferation and division. Owing to the ability of ERK pathway signaling to enhance tumor cell proliferation, ERK inhibitors have been considered potential anticancer drugs [37]. p38 MAPK serves as a regulator of cytokine expression and is in turn activated in response to inflammatory cytokine signaling [38]. As such, p38 is a central regulator of the immune system activity in pathological and physiological contexts. JNKs are stress-activated kinases that control cell survival or apoptotic death in response to diverse stress-related stimuli [37]. MAPK pathway activation has been shown to be a key regulator of mesenchymal stem cell differentiation [39]. Treatment of human DPSCs with LPS induced p38 and ERK activation and downstream IL-8 production [40], and ERK activity is also vital for DMP1, DSPP, and RUNX2 activation within DPSCs [41]. Previous research suggested that sialic acid-binding lectin can induce the intracellular activation of signaling cascades, including the MAPK cascades [42]. We speculated that SA-binding lectin and SA may have something in common, so we tested whether sialic acid (Neu5Ac) affected the MAPK pathway. Herein, we observed no changes in total ERK, JNK, or p38 levels in Neu5Ac-treated DPSCs, whereas p-ERK expression was significantly enhanced in these cells. This suggests that ERK pathway activation is crucial for the SA treatment of DPSCs. We detected no changes in p38 or JNK phosphorylation as a function of Neu5Ac treatment, indicating that these pathways were unaffected by Neu5Ac. To further confirm the relevance of ERK signaling in the context of Neu5Ac-induced DPSC osteo-/odontoblastic differentiation, we treated these cells with the ERK inhibitor cobimetinib. Inhibition of ERK activity reduced DSPP, DMP1, and RUNX2 protein expressions compared to the Neu5Ac group, thus confirming that ERK pathway activation is necessary in order for Neu5Ac to enhance DPSC odontoblastic differentiation.

In summary, the results of the present study revealed that desialylation of DPSC by neuraminidase inhibited osteo-/odontoblastic differentiation while Neu5Ac treatment was able to enhance DPSC osteo-/odontoblastic differentiation. We further determined that ERK signaling was necessary for Neu5Ac to mediate such enhanced differentiation in DPSCs. However, this research only studied the effect of Neu5Ac on the osteo-/odontoblastic differentiation of DPSC. Investigations of Neu5Ac on the development and clinical values of the teeth require further scrutiny.

## Figures and Tables

**Table 1:** RT-PCR primers for the target genes.

Target gene	Primer sequence (5′ to 3′)
DMP1	F: TGAGTGAGTCCAGGGGAGATAA
DMP1	R: TTTTGAGTGGGAGAGTGTGTG C
DSPP	F: TTAAATGCCAGTGGAACCAT
DSPP	R: ATTCCCTTCTCCCTTGTGAC
BSP	F: CCCCACCTTTTGGGAAAACCA
BSP	R: TCCCCGTTCTCACTTTCATAGAT
RUNX2	F:TGGTTACTGTCATGGCGGGTA
RUNX2	R: TCTCAGATCGTTGAACCTTGCTA
GAPDH	F: TGTTCGTCATGGGTGTGAAC
GAPDH	R: ATGGCATGGACTGTGGTCAT

F: forward; R: reverse.

## Data Availability

The data, used during the study, is available from the corresponding author upon reasonable request.
